# Association of Serum Minerals, Vitamin D, Total Protein, and Inflammatory Mediators and Severity of Low Back Pain

**DOI:** 10.31661/gmj.v9i0.1342

**Published:** 2020-10-03

**Authors:** Payman Dadkhah, Seyed Masoud Hashemi, Mehrdad Taheri, Habib Zakeri

**Affiliations:** ^1^Anesthesiology Research Center, Shahid Beheshti University of Medical Sciences, Tehran, Iran; ^2^Noncommunicable Diseases Research Center, Fasa University of Medical Sciences, Fasa, Iran

**Keywords:** Low Back Pains, Minerals, High-Sensitive C-Reactive Protein, Inflammatory Mediators, Vitamin D, Interleukins

## Abstract

**Background:**

Low back pain (LBP) is a multifactorial disorder with multiple etiologies, which are not fully understood. In this study, we aimed to evaluate the relationship between serum levels of minerals, total protein, vitamin D (vit D), and inflammatory mediators with LBP and its severity.

**Materials and Methods:**

This case-control study was derived from the study nested in the Fasa Cohort Study. Overall, 148 individuals with LBP were compared with 150 individuals without LBP. Blood samples were evaluated for serum protein, iron (Fe), aluminum (Al), copper (Cu), phosphorus, vit D, IL-1B, IL-6, high-sensitive C-reactive protein (HS-CRP), and TNF-alpha. Severity of pain was measured with the McGill and Oswestry questionnaires.

**Results:**

The mean age of participants in the case and control groups was 49.2 ± 6.1 and 47.57 ± 5.85 years, respectively. In the case group, 61 patients (48.8%) were male. The mean serum levels of Fe, Al, vit D, Cu, IL-1B, IL-6, HS-CRP, and TNF-alpha were significantly different between case and control groups (P≤0.05). However, there were no significant differences between studied groups in the term of sex and serum phosphorus (P>0.05). Regarding pain severity, age was correlated with McGill score (r=0.18), body mass index with Oswestry (r=0.21), Fe with McGill (r=-0.15) and Oswestry (r=-0.13), protein with McGill (r=0.32) and Oswestry (r=-0.32), Al with McGill (r=0.56) and Oswestry (r=0.45), IL-1B with McGill (r=0.19) and Oswestry (r=0.13), TNF-alpha with McGill (r=0.34) and Oswestry (r=0.26), IL-6 with Oswestry (r=0.13), HS-CRP with McGill (r=0.60) and Oswestry (r=0.46), and vit D was correlated with McGill (r=0.21) and Oswestry scores (r=0.17). Higher Fe (odds ratio [OR]: 0.99), protein (OR: 0.47), Al (OR: 0.11), and vit D levels (OR: 0.97) were protective against LBP (P<0.001). Higher IL-1B (OR: 1.01), TNF-alpha (OR: 1.03), and HS-CRP (OR: 1.0003) presented as risk factors for LBP (P<0.001).

**Conclusion:**

Our study revealed except phosphorous, all the serum levels minerals and inflammatory markers was significantly different in LBP patients compared to healthy individuals. Also, in the LBP patients, serum levels of Fe, total protein, Al, and vit D aside to inflammatory mediators (i.e., IL-1B, TNF-alpha, and HS-CRP) shows a marked association with severity of LBP.

## Introduction

Chronic low back pain (LBP) is among the most common diseases of the 21st century with an estimated annual prevalence of 15 to 45% [1]. It is considered to be the most common reason for medical consultation worldwide [2]. Spinal disorders, especially LBP, increase the cost of health care significantly and may maybe associated with physical and mental disorders even impacting social performances [3,4]. Considering the high costs of pain relief of patients with LBP [5], its correct treatment is importance. On the other hand, treatment of LBP is considered to be extremely difficult [6] as it is mainly considered to be a multifactorial disorder, which are more commonly considered to be psychological and occupational [7]. Although LBP has been widely studied, much remains to be unknown regarding the role of serum biochemical parameters involved in LBP. Evidence demonstrated some patients show clinical improvement when given supplement minerals [8], we hypothesized that some minerals and biochemical parameters in the serum may be playing a part in the pathogenesis of the LBP.

 In this study, using the facilities provided by one of the largest cohort studies in Iran, we evaluated the association between some of the most common serum minerals levels with LBP in a comprehensive manner; moreover, we also evaluated inflammatory markers and vitamin D (vit D) levels as part of the association with LBP.

## Materials and Methods

###  Study Settings

This case-control study was derived of the Fasa Cohort Study (FCS). The FCS was designed to evaluate predisposing factors of common non-transmissible diseases of the rural population in Fasa during a 15 year follow-up period and is part of a larger cohort registry termed the PERSIAN Cohort [9,10]. The participants were invited to partake in this study by health care providers who represent the primary healthcare system at any rural health home and small towns.

In the Iranian model of healthcare network, it is vital that the healthcare providers are chosen from the same city and village as the participants. Every healthcare provider covers an estimated 200-2000 patients and is in direct contact with the people of their specific regions, providing routine and scheduled checkups. The target population of this study was participants aged 35-70 (11097 people), in order to include participants old enough to have been exposed to health risk factors and participants young enough not to be considered as the endpoint of both cardiovascular and non-transmissible diseases. Specifics on the pain cohort protocol has been described previously [11,12].

###  Sample Size Calculation 

 Out of all the patients who were part of the FCS, about 1100 participants reported having LBP for more than 12 weeks. In total, 100 patients were randomly chosen from the 1100 and another 100 patients were randomly chosen and a pilot study was conducted. After primary analysis, the sample size needed to obtain statistical significance was gained. It showed that to obtain a type one error of 0.05 and a power of study of 90%, a minimum of 14% of the 1100 with LBP is needed. Accordingly, final sample size was considered 150 patients.

###  Study Groups

 Considering the main purpose of this study, individuals were specifically selected from the cohort registry. The diagnosis of chronic LBP was done through a questionnaire in the main cohort study that asked if the patient has had back pain for more than 12 weeks. Those who had any other associated diseases or were pregnant were excluded from the study. For the case group among those who had LBP, a total of 150 individuals were randomly selected. Furthermore, for the control group, 150 individuals from the cohort registry were also randomly selected using simple random sampling.

###  Data Collection

To evaluate specifics on LBP, patients would fill out two more questionnaires. The first, was the McGilll Pain Questionnaire [13] to measure the severity of pain associated with their LBP. The second was a LBP specific questionnaire called the Oswestry Disability Index [14]. The Oswestry Disability Index is the most commonly used questionnaire to assess the pain of the lower back. The 10-part questionnaire is designed to assess the limitations of daily activities. Each section is scored between 0-5, and score=5 indicating the highest level of disability. In the end, all the scores are added up and presented in percentages. The measurement of pain was done by a McGilll Pain Questionnaire, which is a detailed questionnaire taking into account different aspects of pain including sensory, affective, and pain levels of the patient. Blood samples taken from patients were kept in the retaining tubes that were specific to each person. Blood sampling was done by a trained personnel dispatched to the center of the cohort located in the Sheshdeh village, Fasa, Iran. A total of 25ml of blood was collected from participants, from which 7ml were stored in clot tubes and the rest were stored in three tubes of 6ml EDTA. Samples were sent to the laboratory located at the Center for Non-Communicable Diseases at the Fasa University of Medical Sciences and were stored within the bio-banks at a temperature of -70°C. Blood samples were evaluated regarding levels of total protein, serum iron (Fe), aluminum (Al), copper (Cu), phosphorus, and vit D. Moreover, inflammatory include levels of IL-1B, IL-6, high-sensitivity C-reactive protein (HS-CRP), and TNF-alpha were also measured.

###  Ethical Consideration

 All individuals gave their written and informed consent to enter the study as part of the cohort registry. The study protocol was also approved by the Institutional Review Board of Fasa University of Medical Sciences (Ethics code: IR.FUMS.REC.1395.100).

###  Data Analysis

 Data analysis was performed using the Stata Software version 12 (). Comparison of normally distributed quantitative data between the case and control groups was done using the Independent T-test. For comparison of qualitative data between groups the Chi-sqaure test was utilized. For evaluation of linear association between level of blood serum parameters and severity of LBP the correlation efficient (r) has been reported. Moreover, for assessment of risk of LBP, the univariate regression analysis was used to evaluate the independent effect of each blood parameter on the occurrence of LBP, reporting the odds ratio (OR) and 95% confidence interval (CI). Data are presented as frequency, percentage, means, and standard deviations (SD), where appropriate. A P-value of less than 0.05 was considered statistically significant.

## Results

 In the case group, two patients were excluded due to incomplete data of questioners. Overall, 148 participants entered the LBP group and 150 individuals entered the control group.

 According to Table-1, patients with LBP were older (49.2 ± 6.1 vs. 47.57 ± 5.85 years, P=0.02), had significantly higher body mass index (BMI; 26.01 ± 4.09 vs. 23.9 ± 2.98; P<0.001), lower levels of serum Fe (569.81 ± 211.63 vs. 664.45 ± 213.7, P<0.001), total protein (7.43 ± 0.55 vs. 7.79 ± 0.84, P<0.001), Al (4.80 ± 0.34 vs. 5.66 ± 0.89, P<0.001), vit D (32.93 ± 16.66 vs. 39.94 ± 20.7, P=0.001), and Cu (23.65 ± 31.23 vs. 30.46 ± 15.68, P=0.023). Patients in the case group had higher levels of IL-1B (80.76 ± 33.26 vs. 67.5 ± 32.22, P=0.001), IL-6 (4.47 ± 9.74 vs. 2.79 ± 1.49, P=0.037), TNF-alpha (45.04 ± 23.77 vs. 28.56 ± 20.51, P<0.001), HS-CRP (4423.42 ± 3583.43 vs. 2486.73 ± 1641.36, P< 0.001). McGill (41.59 ± 5.79 vs. 12.1 ± 4.41, P<0.001), and Oswestry scores (43.64 ± 14.96 vs. 21.89 ± 10.67, P<0.001). Regarding the linear association of severity of pain in LBP, age was correlated with McGill score (r=0.18, P<0.001), BMI was correlated with Oswestry (r=0.21, P<0.001), serum Fe level with McGill (r=-0.15, P<0.05) and Oswestry (r=-0.13, P<0.05), total protein with McGill (r=-0.32, P<0.001) and Oswestry (r=-0.32, P<0.001), Al with McGill (r=0.56, P<0.001) and Oswestry (r=0.45, P<0.001), IL-1B was correlated with McGill (r=0.19, P<0.001) and Oswestry (r=0.13, P<0.05), TNF-alpha with McGill (r=0.34, P<0.001) and Oswestry (r=0.26, P<0.001), IL-6 was correlated with Oswestry (r=0.13, P<0.05), HS-CRP was correlated with McGill (r=0.6, P<0.001) and Oswestry (r=0.46, P<0.001), and vit D was correlated with McGill (r=0.21, P<0.001) and Oswestry (r=0.17, P<0.001). The two pain assessment systems were also strongly correlated (r=0.72, P<0.001). Age was significantly correlated with serum Fe levels (r=-0.12, P<0.05) and HS-CRP levels (r=0.19, P<0.001). BMI was correlated with Al (r=-0.15, P<0.001), IL-6 (r=0.13, P<0.05), and TNF-alpha (r=0.12, P<0.05). Fe serum level were correlated with total protein (r=0.19, P<0.001), Al (r=0.37, P<0.001), Cu levels (r=0.18, P< 0.001), and HS-CRP (r=0.12, P<0.05). Total protein was correlated with Al (r=0.7, P<0.001), Cu levels (r=0.2, P<0.001), IL-1B (r=-0.16, P<0.001), TNF-alpha (r=-0.18, P< 0.001), HS-CRP (r=-0.26, P<0.001), and vitamin D (r=0.13, P<0.05). Al was correlated with copper levels (r=0.21, P<0.001), IL-1B (r=-0.18, P<0.001), TNF-alpha (r =-0.31, P<0.001), HS-CRP (r=-0.29, P<0.001), and vit D (r=0.13, P<0.05). IL-1B was correlated with TNF-alpha (r=0.13, P<0.05). HS-CRP was correlated with vit D (r=0.19, P<0.001, Table-2). In our regression analysis, higher Fe (OR: 0.99, 95% CI=0.996-0.998, P<0.001), total protein (OR: 0.47, 95% CI=0.33-0.67, P<0.001), Al (OR: 0.11, 95% CI=0.06-0.20, P<0.001), and vit D levels (OR: 0.97, 95% CI=0.96-0.99, P<0.001) were protective against LBP. On the other hand, higher levels of IL-1B (OR: 1.01, 95% CI=1.004-1.020, P<0.001), TNF-alpha (OR: 1.03, 95% CI=1.01-1.04, P<0.001), HS-CRP (OR: 1.0003, 95% CI=1.0001-1.0004, P<0.001) presented as significant risk factors for LBP (Table-3).

## Discussion

To the best of our knowledge, this is among the most comprehensive studies evaluating serum biochemical indexes and their relationship with LBP and among the first studies to compare serum levels of Fe, Cu, phosphorus, Al, and total protein aside to inflammatory markers (IL-6, IL-1B, CRP, and TNF-alpha) between those with and without LBP and in relation with degree of perceived pain. We found that LBP was associated with higher serum levels of IL-1B, TNF-alpha, and HS-CRP, and associated with lower serum levels of Fe, total protein, Al, and vit D. Furthermore, severity of pain in LBP to be associated with BMI, serum levels of Fe, total protein, AL, IL-B, IL-1B, TNF-alpha, HS-CRP, and vit D. Although there was not an association between gender and LBP, some studies have found that LBP to be more common among females [15,16]. This may be attributed to pregnancy, which has been shown to be a significant risk factor for LBP, and considering that we excluded pregnant individuals in our study, this maybe the cause as to which females weren’t at higher risk of LBP. Although, some studies [17] have shown a history of pregnancy, rather than current pregnancy, to also be a significant risk factor for LBP. In a large population based study among 92,936 individuals, Heuch *et al.* [18] evaluated the association between BMI and LBP. In their regression model, they found that after adjustment for smoking; leisure time, physical activity, job activity, and BMI was positively associated with higher rates of LBP (OR: 1.17 vs. OR: 1.07 for women and men, respectively). Our study also supported this finding, as we found BMI was significantly higher among those with LBP; moreover, we also found severity of pain positively associated with BMI. Regarding inflammatory mediators and markers, one study in 2013 by Briggs *et al.* [19] using the data from the NHANES registry, found that CRP and obesity highly associated with LBP. Those with elevated CRP had a 1.74 higher chance of reporting LBP, which had an additive effect on risk of LBP when coexisting with obesity. In our study we also found CRP to be significantly associated with LBP in our regression model, although the risk was not high when compared to the mentioned study (OR: 1.003 vs. OR: 1.74). In another study [20], the association between severity of pain and CRP was evaluated among those with chronic LBP. They did not find any statistically significant association in their regression analysis using the visual analogue scale (VAS) for measurement of pain (adjusted OR: 0.87, 95% CI=0.25 - 3). However, in our study, a linear and positive association was observed between pain (using the McGill and Oswestry questionnaires) and HS-CRP levels (r=0.60 and r=0.46, P<0.001, respectively). The interesting result in our study, was the use of HS-CRP for our patients, which allows the detection of minor elevations in CRP. This index has previously been shown was associated with coronary events [21]. Vit D has been rigorously studied regarding its association with pain [22-24]. In a recent study in 2015, among 200 patients with LBP, Lodh *et al*. (6) found that 25-OH vitamin D was significantly low among those with LBP compared to control group. They also found a higher level of CRP in their case group compared to the controls [6]. Other mentioned studies have mostly found that pain was associated with vit D deficiency as well. Studies have attributed this to multiple reasons, including the role of vit D in myopathy, nerve cell regeneration and etc. [25]. Regarding the associaion between minerals and LBP, Vormann *et al*. (8) in an older study evaluated the effects of a 4-week trial of mineral supplementation on 82 patient with LBP. They found that by giving supplement mineral to patients with LBP, pain decreases significantly by 49%. They found the supplement was effective in 76 out of the 82 patients [8]. Although they did not find a significant change in serum levels of calcium, potassium, Fe, Cu, zinc, and phosphorus, serum levels of magnesium shown a significant decrease after receiving supplements. In our study, there was a significant difference between those with and without LBP regarding serum levels of Fe, total protein, Al, and Cu. Moreover, all these minerals (except for Cu and phosphorus) were significantly associated with pain severity in patient with LBP. Our findings bring up the question whether minerals play a causative role in the etiology of LBP, moreover perhaps a modification and supplementation of minerals (specifically Fe, total protein, Al, and vit D) may render therapeutic results in LBP. Our results also showed inflammatory mediators were higher among those with LBP; furthermore, these mediators were positively correlated with pain severity. This shows that perhaps effective control of inflammation may reduce both occurrence and symptoms (pain) in LBP. This study was not without limitation. As our patients were part of the cohort registry, specific lumbar imaging was not performed to rule out any pathologic cause for LBP, and perhaps some patients may have had disk herniation, or degenerative lumbar diseases, which may have caused their LBP. Although the primary objective of our study was to evaluate the relationship between biochemical parameters in the blood and LBP, some factors such as smoking a socio-economic factors may have also contributed to the occurrence of LBP as proposed by some studies [26-28], which were not evaluated in our study. As the study had a case-control design, the nature of the associations between the measured blood related indexes and LBP were not distinguishable and studies with long follow-ups are needed for evaluating causal relationships.

## Conclusion

 We found that except phosphorous, all the serum levels minerals and inflammatory markers was significantly different in LBP patients compared to healthy individuals. Also, in the LBP patients, serum levels of Fe, total protein, Al, and vit D aside to inflammatory mediators (including IL-1B, TNF-alpha and HS-CRP) shows a marked association with severity of LBP.

## Acknowledgement

 The study was part of a larger cohort (the Fasa Cohort Study) and was funded by the Islamic Republic of Iran Ministry of Health grants for development of cohort studies. Authors would like to thank all those at the cohort center and in the Noncomminucable Diseases Research Center who aided in collection of data.

## Conflict of Interest

 Authors have no conflict of interest to declare regarding any part of the study.

**Table 1:** Cross Comparison of Baseline and Blood Related Parameters between Those with Low Back Pain and Those Without Low Back Pain.

**Variables**		**Low back pain (n=148)**	**Control (n=150)**	**Total (n=398)**	**P-value**
Age - yrs		49.20 ± 6.10	47.57 ± 5.85	48.38 ± 6.02	0.020
Sex - no. (%)	Male	61 (48.8)	64 (51.2)	125 (100)	0.80
	Female	87 (50.3)	86 (49.7)	173 (100)	
BMI - Kg/m^2^		26.01 ± 4.09	23.90 ± 2.98	24.95 ± 3.72	<0.001
Fe		569.81 ± 211.63	664.45 ± 213.70	617.45 ± 217.54	<0.001
Copper		23.65 ± 31.23	30.46 ± 15.68	26.90 ± 25.23	0.023
Total protein		7.43 ± 0.55	7.79 ± 0.84	7.61 ± 0.74	<0.001
AL		4.80 ± 0.34	5.66 ± 0.89	5.23 ± 0.80	<0.001
Phosphorus		4.05 ± 3.13	4.16 ± 1.18	4.10 ± 2.36	0.689
IL-1B		80.76 ± 33.26	67.50 ± 32.22	74.08 ± 33.35	0.001
IL-6		4.47 ± 9.74	2.79 ± 1.49	3.63 ± 6.98	0.037
TNF-alpha		45.04 ± 23.77	28.56 ± 20.51	36.74 ± 23.64	<0.001
HS-CRP		4423.42 ± 3583.43	2486.73 ± 1641.36	3448.57 ± 2940.75	<0.001
Vitamin D		32.93 ± 16.66	39.94 ± 20.70	36.46 ± 19.10	0.001
McGil		41.59 ± 5.79	12.10 ± 4.41	26.74 ± 15.63	<0.001
Oswestry		43.64 ± 14.96	21.89 ± 10.67	32.69 ± 16.9 **2**	<0.001

**BMI: **body mass index; **Fe:** Iron; **AL: **aluminum; ** IL:** interleukin; **HS-CRP: **high sensitive C reactive protein *All plus-minus values are means and standard deviations unless stated otherwise.

**Table 2:** Linear Association between Blood Related Parameters and Pain Perception Score in Patients with Low Back Pain. †

	Age	BMI	Fe	T. Pro	AL	Cu	Pho	IL-1B	IL-6	TNF-alpha	HS-CRP	Vit D	McGil	Oswestry
Age	1	-0.005	0.12*	-0.05	-0.02	0.01	-0.05	0.05	-0.01	0.09	0.19**	-0.05	0.18**	0.11
BMI	-0.005	1	-0.07	-0.02	-0.15**	-0.11	0.03	0.06	0.13*	0.12*	0.10	-0.02	0.27**	0.21**
Fe	-0.12*	-0.07	1	0.19**	0.37**	0.18**	-0.004	-0.11	-0.06	0.02	0.12*	-0.08	-0.15*	-0.13*
T. pro	-0.05	-0.02	0.19**	1	0.70**	0.20**	-0.01	-0.16**	-0.10	-0.18**	-0.26**	0.13*	-0.32**	-0.32**
AL	-0.02	-0.15**	0.37**	0.70**	1	0.21**	0.09	-0.18**	-0.11	-0.31**	-0.29**	0.13*	-0.56**	-0.45**
Cu	0.01	-0.11	0.18**	0.20**	0.21**	1	-0.03	-0.05	-0.05	-0.10	-0.01	-0.02	-0.11	-0.11
Pho	-0.05	0.03	-0.004	-0.01	0.09	-0.03	1	-0.01	-0.05	0.001	0.03	-0.01	-0.02	-0.03
IL-1B	0.05	0.06	-0.11	-0.16**	-0.18**	-0.05	-0.01	1	0.07	0.13*	0.03	-0.05	0.19**	0.13*
IL-6	-0.01	0.13*	-0.06	-0.10	-0.11	-0.05	-0.05	0.07	1	0.10	-0.01	-0.03	0.11	0.13*
TNF-alpha	0.09	0.12*	-0.02	-0.18**	-0.31**	-0.10	0.001	0.13*	0.10	1	0.11	-0.09	0.34**	0.26**
HS-CRP	0.19**	0.10	0.12*	-0.26**	-0.29**	-0.01	0.03	0.03	-0.01	0.11	1	-0.19**	0.60**	0.46**
Vit D	-0.06	-0.02	-0.08	0.13*	0.13*	-0.02	-0.01	-0.05	-0.03	-0.09	-0.19**	1	-0.21**	-0.17**
McGil	0.18	0.27	-0.15*	-0.32**	-0.56**	-0.11	-0.02	0.19**	0.11	0.34**	0.60**	-0.21**	1	0.72**
Oswestry	0.11	0.21**	-0.13*	-0.32**	-0.45**	-0.11	-0.03	0.13*	0.13*	0.26**	0.46**	-0.17**	0.72**	1

**BMI: **body mass index; Fe: Iron; T. pro: total protein; AL: aluminum; Cu: copper; pho: phosphorus; IL: interleukin; HS-CRP: high sensitive C reactive protein; **Vit:** vitamin *P<0.05 **P<0.001 †All reported values are correlation coefficients (r). All correlation coefficients have been rounded to the closest 0.01.

**Table 3:** Univariate Regression Analysis for Estimating Risk of Low Back Pain According to Different Blood Related Parameters.

**Variables**	**Odds ratio**	**95% Confidence interval**	**P-value**
**Fe**	0.997	0.996-0.998	<0.001
**Total protein**	0.47	0.33-0.67	<0.001
**AL**	0.11	0.06-0.20	<0.001
**Copper **	0.98	0.96-1.00	0.083
**Vit D**	0.97	0.96-0.99	0.003
**Phosphorus**	0.98	0.88-1.08	0.691
**IL-1B**	1.01	1.004-1.020	0.002
**IL-6**	1.04	0.98--1.10	0.155
**TNF-alpha**	1.03	1.02-1.04	<0.001
**HS-CRP**	1.0003	1.0001-1.0004	<0.001

**Fe:** Iron; **AL: **aluminum; **IL: **interleukin; **HS-CRP: **high sensitive C reactive protein; **Vit:** vitamin
